# The "Newman Policy"

**Published:** 1921-05-14

**Authors:** 


### THE PUBLIC WELL-BEING.

### The "Newman Policy."

It is to their credit that several newspapers, realising the threats levelled in some quarters at the very existence of the Ministry of Health as an effective instrument of national health policy, have made a confession of faith as to the value of what is now commonly called the "Newman policy." It is so called probably because Sir George Newman, Chief Medical Officer of the Ministry, has identified himself with the ideals of the Ministry in an extremely able memorandum, entitled "An Outline of the Principles of Preventive Medicine," and in various other documents which bear the impress of his practical imagination. He must, of course, be regarded as an exponent and not as the author of these ideals, which constitute the *raison d'être* of the State Health Service, and it may be taken for granted that as long as he remains in his present position they will continue so to be.

The national position, as represented in the "Newman policy," was admirably summarised the other day by the medical correspondent of the *Observer*, who pointed out that the profession and practice of medicine, hitherto most intensely individualistic—the doctor waiting for the people to fall ill, and then proceeding to treat them if they call upon him to do so—require in these days a degree of modification, so that, in part at least, the profession may be organised as a national preventive service, fitted for the needs of the people, high and low, rich and poor, and aiming at the creation and maintenance of a healthy nation. For many years past, inevitably, an ever-increasing proportion of the profession has accordingly come to be engaged in such public services as the care of maternity and infancy, the school medical service, the fight against venereal disease, tuberculosis, and other perils to health. Our readers will be aware, for we have frequently had occasion to call their attention to his opinions and advice, that Sir George Newman has been prominently associated with the care of child-life in his official position under the Board of Education; and his past experience under the Board will be of great assistance in enabling him to link up the school medical service with maternity and child-welfare, and with the protection, as the *Observer* correspondent points out, so far imperfectly organised, of the child which is no longer an infant, but not yet a school child —the "pre-school child."

This work, which even the most reactionary person now recognises as an essential part of the State's duty to the nation, is in itself part of a great process of social evolution which no changes of personnel at the Ministry, whether political or expert personnel, can do more than hamper or delay. The medical profession generally is fully alive to the advantages to its own organisation and prospects, as well as to the community in general, of this deliberate, carefully considered, and continuous policy of watching over the health of the people on a principle systematically applied from infancy to adolescence, and of raising preventive medicine to the level of a national responsibility; and what is of even greater importance at the present time, the public mind is conscious at last of the urgent need of a fully-developed State health service. It will certainly never be abolished or starved into uselessness, and we do not think any British Government would now dare to prevent its permanent Health Department from progressing normally along the lines laid down so clearly and convincingly in the memorandum previously referred to.

